# Rapidly patterning micro/nano devices by directly assembling ions and nanomaterials

**DOI:** 10.1038/srep32106

**Published:** 2016-08-26

**Authors:** Na Liu, Feifei Wang, Lianqing Liu, Haibo Yu, Shaorong Xie, Jun Wang, Yuechao Wang, Gwo-Bin Lee, Wen J. Li

**Affiliations:** 1 State Key Lab of Robotics, Shenyang Institute of Automation, Chinese Academy of Sciences, Liaoning 10016, China; 2 School of Mechatronics Engineering and Automation, Shanghai University, Shanghai 200072, China; 3 University of Chinese Academy of Sciences, Beijing, 100049, China; 4 Key Laboratory of Materials for High-Power Laser, Shanghai Institute of Optics and Fine Mechanics, Chinese Academy of Sciences, Shanghai 201800, China; 5 Department of Power Mechanical Engineering, National Tsing Hua University, Hsinchu 30013, Taiwan; 6 Department of Mechanical and Biomedical Engineering, City University of Hong Kong, Kowloon Tong, Hong Kong

## Abstract

The synthesis and assembly of components are key steps in micro/nano device manufacturing. In this article, we report an optically controlled assembly method that can rapidly pattern micro/nano devices by directly assembling ions and nanomaterials without expensive physical masks and complex etching processes. Utilizing this controllable process, different types of device components (e.g., metallic and semiconductor) can be fabricated and assembled in 10–30 seconds, which is far more rapid and cost-effective than any other micro/nano fabrication method.

Micro/nano devices fabrication technologies have advanced to a point where a vast range of insulating, semiconducting and conducting nanocomponents are now available for manufacturing integrated, heterogeneous electronic devices^1^,^2^,^3^. However, there is still the persistent challenge of efficiently assembling these different components into micro/nano-scale devices in a manner similar to the automated fabrication and assembly lines that are widely used for macro-scale manufacturing. For device manufacturing, the most critical steps are component synthesis and assembly. Current micro/nano fabrication methods, such as dielectrophoresis^4^,^5^, atomic force microscopy^6^,^7^,^8^, photolithography^9^, soft lithography^10^,^11^, laser-based printing^12^,^13^,^14^, and electrodeposition^15^,^16^, cannot achieve both high-throughput synthesis and assembly at the same time^17^,^18^. On the other hand, conventional assembly methods, such as dielectrophoresis, atomic force microscopy, and optical tweezers, can offer high-throughput or high-resolution manipulation of nanomaterials, such as DNA, carbon nanotubes (CNTs), and silicon nanowires. Nevertheless, these methods can only assemble devices with prefabricated electrodes, and they pose difficulties for the incorporation of other fabrication methods. Conventional fabrication methods, such as photolithography and soft lithography, have been widely utilized to manufacture micro/nano devices. However, these methods have been criticized for years because of the low patterning flexibility of the photomask fabrication process. Although the laser-based printing methods can fabricate devices in a flexible manner while also enable the fabrication of different types of nanomaterials, the inherent single-point scanning mechanism of these techniques has the drawback of high throughput and efficiency.

Recently, the *optically induced electrokinetics* (OEK) based assembling method has become a viable technology for assembling micro/nano devices. This method has gained wide attention from researchers in the past decade since its great potential in massively manipulating and assembling micro/nano objects, which was firstly reported by Chiou *et al.* in 2005^19^. Numerous works related to OEK based assembling, such as theoretical studies of OEK forces^20^,^21^,^22^, manipulating biological objects (e.g., cells and DNAs)^23^,^24^, and assembling nanowires and nanoparticles for the fabrication of nano-devices^25^,^26^, have been reported to demonstrate the capabilities of the OEK technology. However, a drawback for OEK-based fabrication of functional micro/nano devices is its limitation in patterning highly conductive electrodes. Developed from the reported OEK-based manipulation methods, we have recently revealed an *Optically Controlled Assembly* (OCA) method capable of assembling both ions and nanomaterials to rapidly pattern micro/nano devices using programmable optical images. The OCA method performs micro/nano fabrication in an OCA chip by combining two mechanisms reported by our team in the past: one is the OEK mechanism mentioned above; another is *Optically Induced Electrochemistry* (OEC), which has been shown to be capable of patterning uniform micro-structures using materials ranging from nonconductive polymers to highly conductive metals through direct assembling of molecules or ions^27^,^28^ (but, it cannot manipulate larger size nanostructures such as nanowires and carbon nanotubes). Here, we note that the OCA chip described in this report has a similar structure with traditional OEK chips, but is integrated with a custom-built microfluidic system. That is, the OCA method combines the advantages of OEK, OEC and microfluidics techniques to enable the assembling of micro/nano devices with varying electrical properties, including conductors, semiconductors and insulators, by manipulating targeted ions and nanomaterials (e.g., carbon nanotubes and nanoparticles). We will show in this report that OCA could potentially provide a rapid and cost-effective process for manufacturing micro/nano devices. For example, we have shown that OCA method is capable of patterning gold, silver and copper films within 5–30 seconds, patterning zinc oxide films within 30–60 seconds, and assembling device arrays, such as ZnO-based field-effect transistors (FETs) and CNT-based FETs, within 1–2 minutes.

## Results

### System design

**Figure 1:**
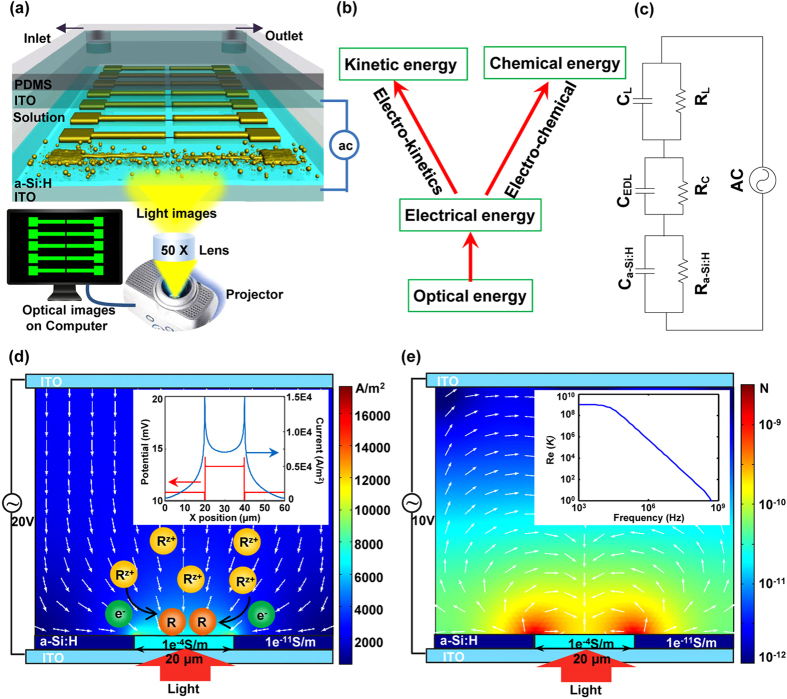
Device structures and mechanisms for assembling ions and nanomaterials. (**a**) Illustration of the OCA experimental system. (**b**) Two mechanisms for assembling ions and nanomaterials. (**c**) Equivalent circuit model of the OCA chip. (**d**) Distribution (color) and directions (arrows) of current in a 100-mM Zn(NO_3_)_2_ solution when a 10-kHz, 20-V_pp_ AC signal is applied. The inserted figure shows the potential and current on the EDL. (**e**) Distribution (color) and direction (arrows) of DEP force exerted on the SWNTs in a chip when a 100-kHz, 10-V_pp_ AC signal is applied. The inserted figure shows Re(*K*) of the SWNTs at different frequencies.

The experimental system consists of three main parts (Fig. 1a and Figure s1). The first part is the image design system that generates the “virtual” electrodes, which includes one personal computer equipped with a commercial graphic animation software (Adobe Flash 11, Adobe, USA) used to design images and a digital liquid crystal display (LCD) projector (Sony VPL-F400X, Japan) and a condenser lens (Nikon 50X/0.55) used to project light images onto the OCA (optically controlled assembly) chip. The second part is the real-time monitoring system, which includes a charge-coupled device (CCD) camera attached to a microscope. The third part of the experimental system is the OCA chip itself, which consists of five layers: the polydimethylsiloxane (PDMS) layer designed for transferring solution, the upper indium tin oxide (ITO) electrode, the double adhesive tape layer used to form the solution chamber, and the bottom photoconductive film (a-Si:H) deposited ITO electrode.

When fabricating devices by OCA, electrolyte solutions used for patterning different components are automatically and orderly injected into the OCA chamber through the PDMS microfluidic channels. Then, an alternating current (AC) is applied across the top and the bottom ITO electrodes. Simultaneously, the designed optical images are projected onto the photoconductive layer. When optical images are projected onto the photoconductive a-Si:H substrate, the electrical conductivity of the irradiated regions can increase by several orders of magnitude owing to the photo-generated electro-hole pairs. For example, when projecting optical images with a RGB value of (0, 255, 0) onto the substrate, the conductivity of the a-Si:H can increase from 10^−11^ S/m to 10^−5^ S/m. With this technique, “virtual” electrodes can be created directly on the a-Si:H surface by projecting customized optical images. Except the negligible optical scattering around the projected images’ boundary on the a-Si:H layer, the shape and size of the “virtual” electrodes are completely defined by the optical images. In the solution atop these “virtual” electrodes, two optional mechanisms can be applied (Fig. 1b). Optically controlled electrochemistry, which converts optical and electrical energy into chemical energy, can be used to target ions in the solution layer and assemble them into arbitrary micro/nano structures through a series of chemical reactions. Alternatively, optically controlled electrokinetics, which converts optical and electrical energy into kinetic energy, can be utilized to manipulate and assemble suspended objects at micro/nano-scale in a highly efficient manner.

### Mechanisms and applications of patterning structures

As mentioned above, this method presents two different mechanisms of assembling ions and nanomaterials. The choice of fabrication mechanism depends on the required components. Both mechanisms can be selected and controlled by adjusting the solution composition and applying different AC signals.

As shown in Fig. 1c, the OCA chip can be treated as a series circuit of the a-Si:H film, the electrical double layer (EDL) on the a-Si:H/solution interface, and the solution layer. In the circuit, *R*_a-Si:H_ and *C*_a-Si:H_ denote the resistance and the capacitance of a-Si:H, respectively; *C*_*EDL*_ is the capacitance of the EDL; *R*_C_ represents the resistance induced by electrochemical reactions on the a-Si:H/solution interface; and *R*_L_ and *C*_L_ denote the resistance and capacitance of the solution layer, respectively. Among them, the *C*_EDL_ and *R*_C_ are difficult to calculate because they are interconnected and affected by multiple factors, such as the parameters (amplitude and frequency) of AC voltage and solution composition. Nevertheless, the equivalent circuit model is capable of expressing the assembly mechanisms and processes after reasonable simplifications according to the experimental conditions. Based on the Gouy-Chapman-Stern model, the EDL consists of the Stern layer and the diffuse layer, but the effect of the diffuse layer is negligible when ion concentration in solution is greater than 100 mM. Therefore, *C*_EDL_ can be approximated by the capacitance of the Stern layer (*C*_S_) as following equation^19^,^29^,^30^,


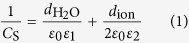


where 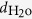 is the diameter of a water molecule, *d*_ion_ is the diameter of the hydrated ions, *ε*_0_ is the vacuum permittivity, *ε*_1_ ≈ 6 is the relative permittivity of H_2_O in the inner Helmholtz layer, and *ε*_2_ ≈ 30 is the relative permittivity of hydrated ions across the inner and outer Helmholtz layers. Assuming the potential drop across the EDL is *ϕ*_0_, which can be evaluated by the following equation,


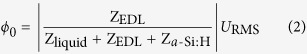


where the 
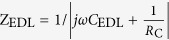
, *Z*_liquid_ and *Z*_a-Si:H_ denote the impedances of the EDL, the solution layer and the a-Si:H substrate, respectively, and *U*_RMS_ is the time-averaged amplitude of the applied AC signal. Obviously, *ϕ*_0_ is determined by solution conductivity, *C*_EDL_, *R*_C_, AC frequency and amplitude. Assuming the maximum equilibrium potential of the electrochemical reactions on the a-Si:H/interface layer is *ϕ*_1_, the value of which is unknown and dependent on the reaction type, solution composition, and electrode material. When *ϕ*_0_ > *ϕ*_1_, the equilibrium on the a-Si:H surface is broken, the net ion reactions occur and the designed products are generated. When *ϕ*_0_ < *ϕ*_1_, there are no net chemical reactions and products. Thus, the occurrence of ion reaction and formation of designed components are decided by *ϕ*_0_. Considering a critical state, when *ϕ*_0_ is infinitely approaching to *ϕ*_1_ but no net chemical reactions occur, the *Z*_EDL_ can be simplified as 1/(j*ωC*_EDL_), and *ϕ*_0_ can be approximately calculated using Eq. (2).

Based on the parameters and equivalent circuit above, the distribution of electric potential and current in a 100-mM Zn(NO_3_)_2_ solution was simulated using a finite element method (FEM) software package (Multiphysics, COMSOL AB, Sweden). As shown in Fig. 1d, a two-dimensional model was built, the projected light image was set to a size of 20 μm, the height of the solution layer was 50 μm, and a 20-V_PP_, 10-kHz voltage was applied. The color table denotes the distribution of the current density, and the arrows indicate the current direction. As shown in the Figure, the majority of current flows through the illuminated a-Si:H areas. The potential and current density on the EDL shown in the inset of Fig. 1d indicate that the EDL layer a top the illuminated a-Si:H surface has much larger potential and current density than the EDL layer atop the non-illuminated a-Si:H surface. Based on Eq. (2), potential on the EDL (*ϕ*_0_) decreases with the increase of frequency when other parameters are constant. Thus, there exists a frequency range, within which the *ϕ*_0_ is large enough to activate the chemical reactions. In addition, the ion chemical reactions are confined to regions directly above these “virtual” electrodes and the micro/nano structures are formed in the same pattern as the projected light images. As with traditional electrochemical reactions, various products and structures can be designed and obtained on the “virtual” electrodes by adjusting solution composition. In this work, metallic and semiconductor films with optically controllable shapes have been assembled using OCA method.

Based on the components required for assembly, conditions conducive for electrochemical reactions are sometimes undesirable for OCA. When nanomaterials are to be manipulated and assembled rather than ions, the optically controlled electrokinetics forces are used. In these cases, chemical reactions should be avoided because they often generate unwanted products such as bubbles, which will block the assembly of targeted nanomaterials. To ensure that no net chemical reactions occur and allow optically controlled electrokinetics, *ϕ*_0_ should be smaller than *ϕ*_1_, and the solution should have low ion concentration and low conductivity. Because no net electrochemical reactions occur with this mechanism, the *R*_C_ in the equivalent circuit is infinite and its influence can be ignored. The effect of the Stern layer in the EDL is also negligible, and *C*_EDL_ can be approximated by the following equation^22^,


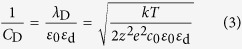


where *λ*_D_ denotes the thickness of the diffuse layer, and *K*, *T*, *e*, *z*, and *c*_0_ are the Boltzmann constant, temperature, electron charge, ion valence, and ion concentration, respectively. Under these conditions, most of the applied voltage is shifted to the solution layer, generating a non-uniform electric field in the solution atop the “virtual” electrodes. Electrokinetic phenomena such as dielectrophoresis (DEP)^19^, AC electroosmosis (ACEO)^22^, and AC electrothermal (ACET)^31^ effects can then be induced. Among them, the DEP force is most frequently used to manipulate and assemble nanomaterials because it enables selectively assembling objects. The DEP force is directly exerted on objects, and its value depends on parameters of both the objects and the liquid media. However, ACEO and ACET forces are exerted on liquid; fluid flow can drive objects in the liquid to move. Take the manipulation of single-walled nanotubes (SWNTs) using DEP force as an example. Based on an elongated ellipsoid model, the DEP force is given by 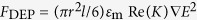, where *r* and *l* are nanotube radius and length, respectively; Re(*K*) is the real component of the Clausius-Mossotti factor (*K*), and ∇*E*^2^ is the gradient of the squared electric field. The Clausius-Mossotti factor is a function of the permittivity and conductivity of the manipulated objects and the media. For an elongated object with its long axis aligned along the electric field, *K* is given by^25^,^30^,^32^


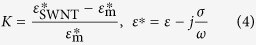


Here, a FEM software package was used to calculate the approximate field gradients and the corresponding forces exerted on suspended SWNTs (*r* = 1 nm, *l* = 10 μm), assuming most SWNTs are semiconductors and have a conductivity of 10^5^ S/m and a relative permittivity of 2.5 32. The inset of Fig. 1e shows the Re(*K*) of SWNTs under different frequencies and indicates a positive DEP force transports the SWNTs toward the strong regions of electric field. The color and the arrows in Fig.1e represent the distribution and the direction, respectively, of DEP forces exerted on the SWNTs in the solution layer. As shown in Fig. 1e, the DEP forces transport the SWNTs to the illuminated a-Si:H areas. In addition, the DEP force increases as the SWNTs move closer to the surface. Because the “virtual” electrodes are defined by the projected light images, which can be dynamically changed, the regions where SWNTs can be assembled are also defined by the projected light images.

### Patterning of ZnO-based devices

**Figure 2:**
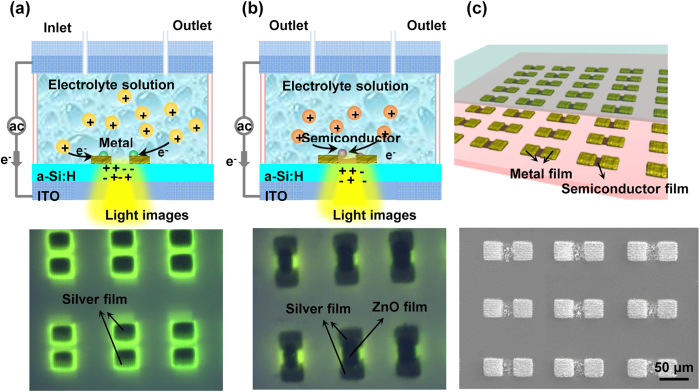
Process of assembling ZnO-based devices. (**a**) The upper and lower figures show the illustrated and experimental results of patterning metallic films, respectively. (**b**) The upper and lower figures are the illustrated and experimental results of assembling ZnO film with the pre-patterned silver films, respectively. (**c**) The illustrated and experimental results of assembled devices.

As mentioned above, specific chemical reactions can be leveraged to assemble targeted ions into designed structures. Here, an efficient fabrication process for ZnO-based devices has been demonstrated by patterning and assembling metallic electrodes on ZnO films (Fig. 2). ZnO is an n-type semiconductor with a direct wide bandgap of ~3.4 eV; it is used for a wide variety of applications in many fields^33^,^34^. The assembly process of ZnO-based devices consists of only two steps: 1) the electrolyte solution for fabricating metal electrodes is transferred into the chamber, and metal electrodes with customized shapes and sizes are fabricated on the a-Si:H surface by a metal electrodeposition process (Fig. 2a); 2) the original solution is then replaced with a zinc nitrate solution to pattern ZnO films and assemble devices (Fig. 2b). Once the ZnO films have been patterned with the prefabricated silver electrodes, fabrication of the ZnO-based devices is complete. The SEM images illustrate a good connection between the ZnO films and the silver films (Fig. 2c).

Specifically, various types of metal electrodes can be fabricated using the OCA process. For example, solutions containing Au^3+^, Cu^2+^ and Ag^+^ have been used to assemble gold, copper and silver electrodes, respectively, through the following processes:


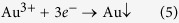



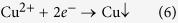



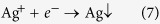


**Figure 3:**
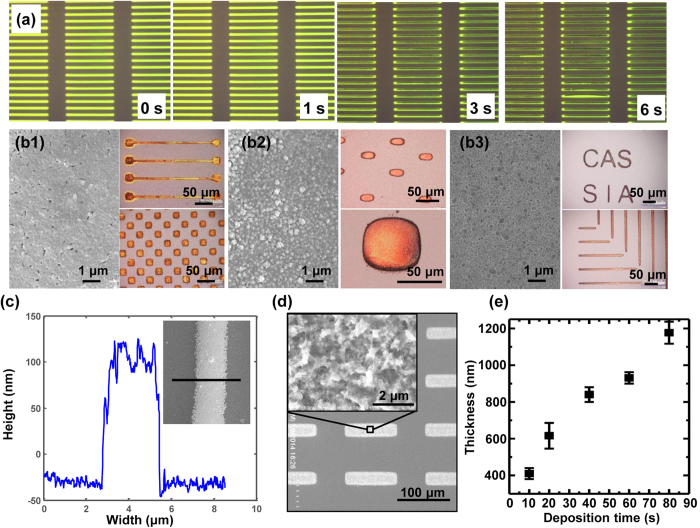
Patterning results of metallic films and ZnO films using the OCA method. (**a**) The patterning process of gold electrodes using the OCA method. (**b**) SEM and optical images of patterned gold (b1), copper (b2), and silver (b3) metallic films. (**c**) Smallest width achieved by the OCA method. (**d**) SEM image of patterned ZnO films. (**e**) The relationship between deposition time and thickness of ZnO films.

During the metallic electrode patterning process, metal ions are first reduced to atoms, which then crystallize and grow to be metallic structures on the “virtual” electrodes. Owing to the confinement of the chemical reactions to the “virtual” electrodes, the shapes of the patterned structures are completely defined by the projected light images (Fig. 3a). Utilizing the OCA process, uniform gold films with a thickness of ~500 nm were fabricated in 5 seconds in a 20-mM AuHCl_4_ solution under a 20-V_pp_, 20-kHz square-wave current (Fig. 3b1). Copper films with a thickness of ~1 μm were deposited in 10 seconds using a 50-mM CuSO_4_ solution under a 20-V_pp_, 20-kHz square-wave current (Fig. 3b2). Silver films with a thickness of ~1 μm were fabricated in 30 seconds using a 100-mM AgNO_3_ solution under a 10-V_pp_, 50-kHz sinusoidal current (Fig. 3b3). The smallest feature width achieved by the OCA method in our present system was approximately 2.7 μm (Fig. 3c), which could be further improved by incorporating a higher-resolution projector and lens. Furthermore, the thickness of the patterned metallic films is directly proportional to the deposition time if the same AC voltage is applied. This can be elaborated by the Faraday theory 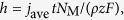 where *j*_ave_ is the time-averaged electric current density, *t* is the deposition time, *N*_M_, *ρ* are the molar mass and density of the deposited film, *z* is the ion valence of the metal ions, and *F* is the Faraday constant. Owing to the crystallization and growth process, the deposited metallic films possess excellent electrical conductivity. For example, the OCA assembled silver films have been demonstrated a high electrical conductivity of 2 × 10^7^ S/m^27^.

After synthesis of the metallic electrodes, a 100-mM zinc nitrate solution was injected into the chamber to assemble the semiconductor components. A 20-V_pp_, 20-kHz sinusoidal current was applied. The n-type ZnO films were deposited through the following processes:


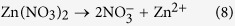



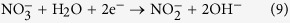



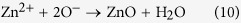


The XPS spectra shows that the deposited films consist of Zn and O elements, and the detailed morphology shown by SEM and XRD indicates a porous and amorphous structure (Fig. 3d, Figure s2). The amorphous structures were mainly caused by the low deposition temperature. It has been reported that the morphology and crystallization of electrodeposited ZnO are significantly influenced by the deposition temperature; ZnO films with well-defined preferential orientations can be deposited when the bath temperature exceeds 343 K^35^. Those results suggest that ZnO films with oriented growing directions could also be deposited using the OCA process by increasing the solution temperature. In addition, ZnO film thickness also increases with increasing deposition time, but not linearly as it does with the deposition of metallic films (Fig. 3e). This is because the resistance of the metallic films is negligible and has no obvious effect on the faradic current during the deposition process. However, because ZnO films are semiconductors, film resistance will increase obviously with increasing thickness, leading to decreasing faradic current and growth rate of the ZnO films.

**Figure 4:**
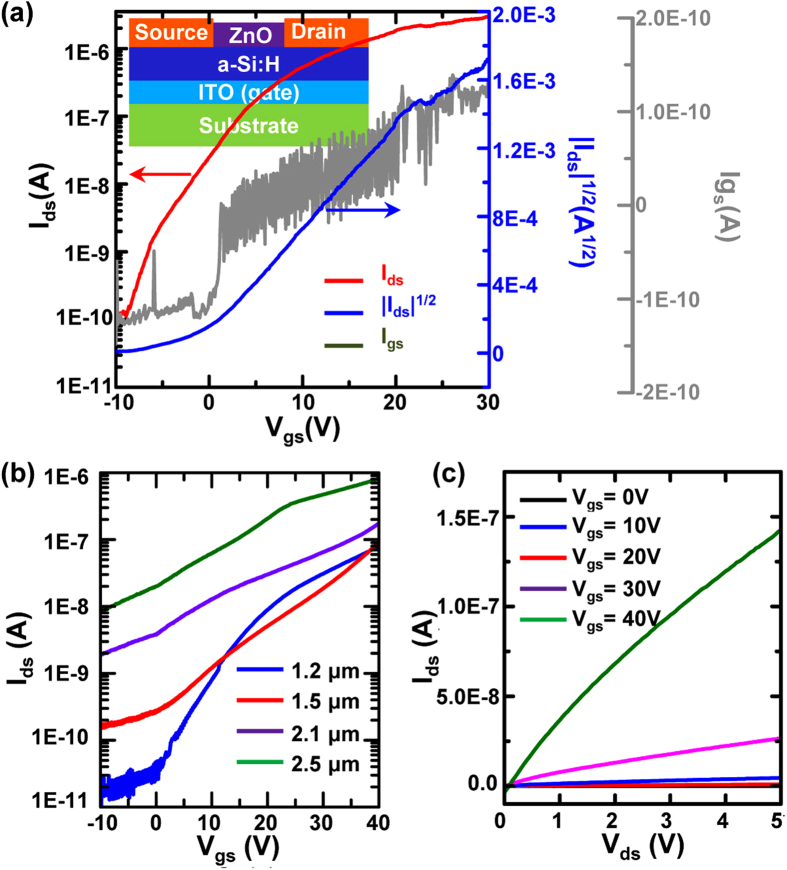
The structure and properties of assembled ZnO-based FETs. (**a**) Structure and transfer characteristics of a ZnO-based FET. (**b**) Transfer characteristics of FETs assembled with ZnO of different thicknesses. (**c**) Output characteristics of FETs assembled by a ZnO film with a thickness of 1.2 μm.

The assembled metallic films and ZnO films could serve as devices such as optical or chemical gas sensors owing to the intrinsic properties of ZnO. Even with amorphous structures, the devices also function as thin film field-effect transistors (FETs), as shown in Fig. 4a, where the ZnO films act as n-type semiconductors, the metallic films act as source and drain electrodes, the a-Si:H substrate functions as a dielectric film, and the ITO electrodes function as gate electrodes. The transfer and output characteristics of the ZnO-based FETs were measured using a semiconductor parameter analyzer (Agilent 4155C, USA) on an analytical probe station (Everbeing DB-8, Taiwan). The ZnO films were measured to have a thickness of ~1.3 μm, length of 50 μm, and width of 27 μm. As shown in Fig. 4a, the transfer characteristic curve demonstrates a high on/off ratio of 10^4^ when *V*_gs_ (the voltage between the gate electrode and source electrode) changes from −10 V to 30 V. As shown, *I*_ds_ increases as *V*_gs_ increases, which indicates the ZnO-based FETs are n-type. The measured *I*_gs_ suggests a very small leakage current. Based on the slope of *I*_ds_^1/2^ versus *V*_gs_, the saturation electron mobility can be determined according the following equation^36^.


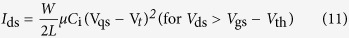


where *C*_i_ is the capacitance per unit area of the insulator (*ε*_r_ = 11), which is 9.4 × 10^−6^ F/m^2^, and *V*_th_ is the threshold voltage, which is −1.1 V. The calculated mobility value for *V*_gs_ ranging from 2.9 V to 11.8 V is 39.4 cm^2^/(V · s).

Because of the image-defined patterns and time-dependent thickness, the device performance can be easily controlled by adjusting the optical images and the deposition time for patterning semiconductor films. To investigate how the thickness of ZnO films affects the properties of assembled FETs, ZnO films with the same shape (75 μm in length and 24 μm in width) but different thicknesses were deposited. Both the transfer characteristics (Fig. 4b) and output characteristics (Figure s3 and Fig. 4c) of the FETs were measured. To examine the output characteristics, the drain current (*I*_ds_) was measured with a fixed gate voltage (*V*_gs_) from 0 V to 40 V at steps of 10 V, and the drain voltage (*V*_ds_) was scanned from 0 to 5 V. To measure the transfer characteristics, the gate voltage (*V*_gs_) was scanned from −10 V to 30 V, and the drain voltage was fixed at 5 V. As shown in Fig. 4b, the measured results suggest that as the thickness of ZnO films increases, the on/off ratio of the FETs will decrease, but the output current will increase. This phenomenon can be illustrated by the working principle of FETs; that is, the resistance of the semiconductor film can be adjusted by the electric field induced by *V*_gs_. When the semiconductor is n-type, the main charge carriers are electrons; thus, applying a positive *V*_gs_ would increase the number of electrons. As a result, both the conductivity and current (*I*_ds_) of the semiconductor films would increase. When the semiconductor is p-type, the main charge carriers are holes; then, a negative *V*_gs_ could be used to increase the number of holes and the conductivity. Based on the Ohm’s law, increasing the thickness would also decrease the electrical resistance. Therefore, the effect of the electric field on semiconductor film resistance may be eclipsed by increasing the thickness of the semiconductor films, resulting in a decreased on/off ratio. Ideally, the semiconductor film should be thin enough to produce a large on/off ratio, but thinner films may lead to smaller output currents. However, a large output current is usually required to drive subsequent devices in most applications. Thus, device sizes should be designed specifically for each unique application.

### Assembly of SWNT-based devices

**Figure 5:**
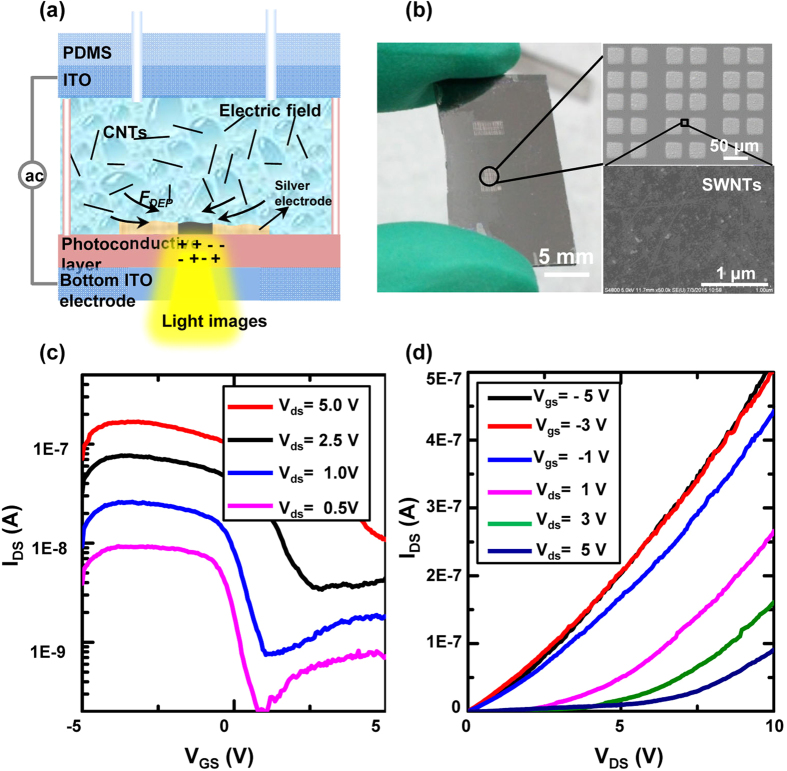
The assembly of SWNT-based FETs. (**a**) Illustration of SWNT assembly process. (**b**) Optical and SEM images of the SWNT-based FETs. (**c**) Transfer characteristics of a SWNT-based FET under different drain voltages. (**d**) Output characteristics of the FET.

In addition to enabling the assembly of n-type FETs through the direct patterning of ZnO films with silver electrodes, the OCA method also enables the parallel manipulation and assembly of nanomaterials. Here, the fabrication of SWNT-based devices is demonstrated through the assembly of SWNTs with silver electrodes. To assemble the SWNTs, silver electrodes were patterned using an AgNO_3_ solution, and then, the SWNT solution (supplementary note 2) was injected into the chamber. An alternating signal with an amplitude of 10 V_pp_ and a frequency of 100 kHz was applied. As previously discussed, the image-patterned “virtual” electrodes could generate a non-uniform electric field and DEP forces on the SWNTs. When projecting light images across the prefabricated silver electrodes, the SWNTs in solution were transported toward and deposited onto the illuminated a-Si:H surface by the DEP forces (Fig. 5a). Figure 5b shows the optical and SEM images of the assembled SWNTs and silver films. Utilizing the same measuring method, the transfer and output characteristics of the SWNT-based FETs (length of 14 μm and width of 26 μm) were obtained. As shown in Fig. 5c, transfer characteristics under different drain voltages were obtained when the gate voltage (*V*_gs_) scanned from −5 V to 5 V. The curves indicate that SWNT-based FETs exhibit p-type properties. Under a drain voltage of 0.5 V, the FETs show an on/off ratio of 100. However, the on/off ratio decreases as the applied drain voltage increases. Figure 5d shows the output characteristics when drain voltage scanned from 0 V to 10 V under different gate voltages. Compared with the patterned ZnO-based transistors, the SWNT-based transistors show a faster on/off rate and a smaller work voltage.

## Discussion

An optically controlled assembly method incorporating synthesis and assembly of multifunctional components for manufacturing micro/nano devices has been demonstrated in this work. Utilizing this method, the rapid patterning of metallic and semiconductor films *in situ* can be realized in tens of seconds through the assembly of either target ions or nanomaterials. This method leverages computer-designed optical images that can be dynamically reshaped rather than photomasks that have fixed patterns, and thus enables dynamic control over both device shape and size in real-time. By applying a microfluidic technique, different solutions were transferred into the OCA chip for orderly assembly into different device components. While only the patterning processes for ZnO-based and SWNT-based devices have been demonstrated, the assembly mechanisms of the OCA method are capable of manufacturing many other types of devices.

## Additional Information

**How to cite this article**: Liu, N. *et al.* Rapidly patterning micro/nano devices by directly assembling ions and nanomaterials. *Sci. Rep.* **6**, 32106; doi: 10.1038/srep32106 (2016).

## Supplementary Material

Supplementary Information

Supplementary Video 1

Supplementary Video 2

Supplementary Video 3

## References

[b1] Cao J. *et al.* Carbon nanotube/cds core–shell nanowires prepared by a simple room-temperature chemical reduction method. Adv. Mater. 16, 84–87 (2004).

[b2] Fan P. *et al.* An invisible metal-semiconductor photodetector. Nat. Photonics 6, 380–385 (2012).

[b3] Wang Q. H., Kalantar-Zadeh K., Kis A., Coleman J. N. & Strano M. S. Electronics and optoelectronics of two-dimensional transition metal dichalcogenides. Nat. Nanotechnol. 7, 699–712 (2012). 23132225 10.1038/nnano.2012.193

[b4] Shim H. C., Kwak Y. K., Han C.-S. & Kim S. Effect of a square wave on an assembly of multi-walled carbon nanotubes using AC dielectrophoresis. Physica. E 41, 1137–1142 (2009).

[b5] Vijayaraghavan A. *et al.* Ultra-large-scale directed assembly of single-walled carbon nanotube devices. Nano Lett. 7, 1556–1560 (2007). 17488050 10.1021/nl0703727

[b6] Nam K. *et al.* Single-step electropolymerization patterning of a polypyrrole nanowire by ultra-short pulses via an AFM cantilever. Nanotechnology 22, 225303 (2011). 21464524 10.1088/0957-4484/22/22/225303

[b7] Xie X. N., Chung H. J., Sow C. H. & Wee A. T. S. Nanoscale materials patterning and engineering by atomic force microscopy nanolithography. Mat. Sci. Eng. R 54, 1–48 (2006).

[b8] Garcia R., Knoll A. W. & Riedo E. Advanced scanning probe lithography. Nat. Nanotechnol. 9, 577–587 (2014). 25091447 10.1038/nnano.2014.157

[b9] Ok J. G., Kwak M. K., Huard C. M., Youn H. S. & Guo L. J. Photo-roll lithography (PRL) for continuous and scalable patterning with application in flexible electronics. Adv. Mater. 25, 6554–6561 (2013). 24014277 10.1002/adma.201303514

[b10] Sessolo M. *et al.* Easy-to-fabricate conducting polymer microelectrode arrays. Adv. Mater. 25, 2135–2139 (2013). 23417987 10.1002/adma.201204322

[b11] Qin D., Xia Y. & Whitesides G. M. Soft lithography for micro-and nanoscale patterning. Nat. Protoc. 5, 491–502 (2010). 20203666 10.1038/nprot.2009.234

[b12] Son Y. *et al.* Nanoscale electronics: digital fabrication by direct femtosecond laser processing of metal nanoparticles. Adv. Mater. 23, 3176–3181 (2011). 21618292 10.1002/adma.201100717

[b13] Lu W.-E. *et al.* Femtosecond direct laser writing of gold nanostructures by ionic liquid assisted multiphoton photoreduction. Opt. Mater. Express 3, 1660 (2013).

[b14] Zeng H. *et al.* High-resolution 3D direct laser writing for liquid-crystalline elastomer microstructures. Adv. Mater. 26, 2319–2322 (2014). 24421068 10.1002/adma.201305008

[b15] Cherevko S. & Chung C.-H. Direct electrodeposition of nanoporous gold with controlled multimodal pore size distribution. Electrochem. Commun. 13, 16–19 (2011).

[b16] Pascall A. J. *et al.* Light-directed electrophoretic deposition: a new additive manufacturing technique for arbitrarily patterned 3D composites. Adv. Mater. 26, 2252–2256 (2014). 24532281 10.1002/adma.201304953

[b17] Moonen P. F., Yakimets I. & Huskens J. Fabrication of transistors on flexible substrates: from mass-printing to high-resolution alternative lithography strategies. Adv. Mater. 24, 5526–5541 (2012). 22887056 10.1002/adma.201202949

[b18] Yeo J. *et al.* Next generation non-vacuum, maskless, low temperature nanoparticle ink laser digital direct metal patterning for a large area flexible electronics. PLoS One 7, e42315 (2012). 22900011 10.1371/journal.pone.0042315 PMC3416833

[b19] Chiou P. Y., Ohta A. T. & Wu M. C. Massively parallel manipulation of single cells and microparticles using optical images. Nature 436, 370–372 (2005). 16034413 10.1038/nature03831

[b20] Liang W. *et al.* In Nano/Micro Engineered and Molecular Systems (NEMS), 2011 IEEE International Conference on. 825–830 (IEEE).

[b21] Hwang H. & Park J. K. Rapid and selective concentration of microparticles in an optoelectrofluidic platform. Lab Chip 9, 199–206 (2009). 19107274 10.1039/b811740c

[b22] Pei-Yu C., Ohta A. T., Jamshidi A., Hsin-Yi H. & Wu M. C. Light-actuated AC electroosmosis for nanoparticle manipulation. J. Microelectromech. Syst. 17, 525-53 1 (2008).

[b23] Lin Y. H., Chang C. M. & Lee G. B. Manipulation of single DNA molecules by using optically projected images. Opt. Express 17, 15318–15329 (2009). 19688010 10.1364/oe.17.015318

[b24] Lin Y.-H. & Lee G.-B. An integrated cell counting and continuous cell lysis device using an optically induced electric field. Sensor Actuat. B-Chem. 145, 854–860 (2010).

[b25] Jamshidi A. *et al.* Dynamic manipulation and separation of individual semiconducting and metallic nanowires. Nat. Photonics 2, 86–89 (2008). 19789729 10.1038/nphoton.2007.277 PMC2752982

[b26] Lee M.-W., Lin Y.-H. & Lee G.-B. Manipulation and patterning of carbon nanotubes utilizing optically induced dielectrophoretic forces. Microfluid. Nanofluid. 8, 609–617 (2010).

[b27] Liu N. *et al.* Optically-controlled digital electrodeposition of thin-film metals for fabrication of nano-devices. Opt. Mater. Express 5, 838–848 (2015).

[b28] Liu N. *et al.* 3-D non-uv digital printing of hydrogel microstructures by optically controlled digital electropolymerization. J. Microelectromech. S. 24, 2128–2135 (2015).

[b29] Hamann C. H., Hamnett A. & Vielstich W. Electrochemistry. (Wiley-VCH, 1998).

[b30] Morgan H. & Green N. G. AC electrokinetics: colloids and nanoparticles. (Research Studies Press, 2003).

[b31] Liang W. *et al.* Rapid assembly of gold nanoparticle-based microstructures using optically-induced electrokinetics. Opt. Mater. Express 4, 2368 (2014).

[b32] Dimaki M. & Bøggild P. Dielectrophoresis of carbon nanotubes using microelectrodes: a numerical study. Nanotechnology 15, 1095–1102 (2004).

[b33] Xu S. & Wang Z. L. One-dimensional ZnO nanostructures: Solution growth and functional properties. Nano Res. 4, 1013–1098 (2011).

[b34] Kim K. *et al.* Patterning of flexible transparent thin-film transistors with solution-processed zno using the binary solvent mixture. Adv. Funct. Mater. 21, 3546–3553 (2011).

[b35] Otani S., Katayama J., Umemoto H. & Matsuoka M. Effect of bath temperature on the electrodeposition mechanism of zinc oxide film from zinc nitrate solution. J. Electrochem. Soc. 153, C551–C556 (2006).

[b36] Pal B. N., Trottman P., Sun J. & Katz H. E. Solution-deposited zinc oxide and zinc oxide/pentacene bilayer transistors: High mobility n-channel, ambipolar, and nonvolatile devices. Adv. Funct. Mater. 18, 1832–1839 (2008).

