# Supplementary material for: Rapidly patterning micro/nano devices by directly assembling ions and nanomaterials
Source: Sci Rep. 2016 Aug 26;6:32106. doi: 10.1038/srep32106 (PMC4999812; doi:10.1038/srep32106)
Supplement: Supplementary Information [file srep32106-s1.pdf]

# Rapidly patterning micro/nano devices by directly assembling ions and nanomaterials

Na Liu[1,2], Feifei Wang[1,3], Lianqing Liu[1,*], Haibo Yu[1], Shaorong Xie[2], Jun Wang[4], Yuechao Wang[1], Gwo-Bin Lee [1,5], and Wen J. Li [1,6,*]

***Note 1: Assemble process of ODE chip***

The PDMS layer with patterned fluidic channels (0.5 mm × 0.5 mm) was fabricated by a process of mold replication and heat-curing. Specifically, cast a pre-polymer solution containing PDMS and a curing agent (Sylgard® 184, Dow Corning, USA) at a ratio of 10:1 on a pre-fabricated PMMA substrate firstly, and then cure the PDMS at 70 °C for 6 hours to replicate the fabricated micro-ridges. Two holes with diameters of 1 mm were drilled in the upper ITO glass. Then, bond the PDMS layer with the upper ITO glass via an oxygen plasma treatment process. The a-Si:H film was deposited on the bottom ITO glass through a plasma-enhanced chemical vapor deposition (PECVD) process. Finally, attach the upper ITO glass with the bottom ITO glass using a double adhesive layer. A micro-chamber (6 mm × 10 mm × 50 μm) was formed upon the a-Si:H substrate in the assembled OCA chip. Solution, such as silver nitrate solution, zinc nitrate solution and deionized water, can be injected into the chamber using a syringe pump.

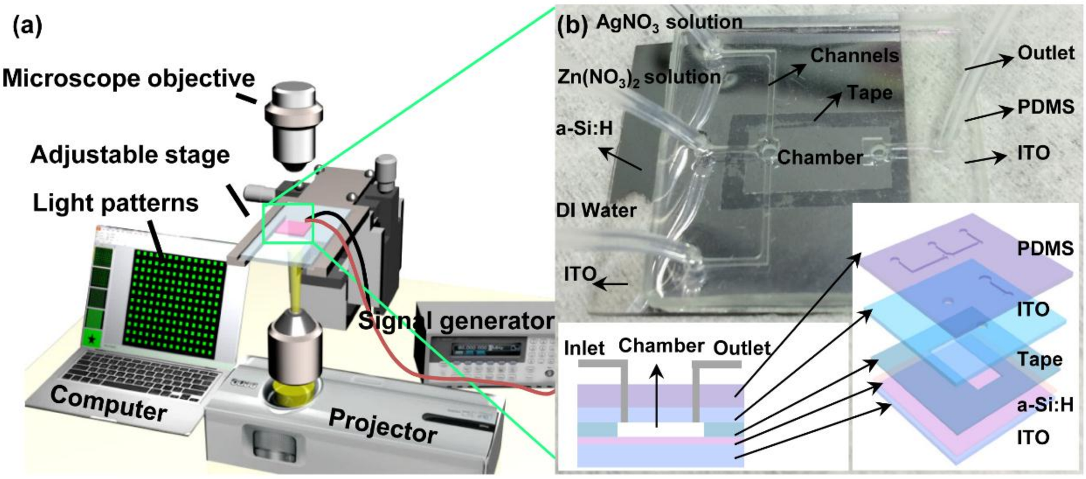


**Figure s1.** (a) Illustration of the OCA experimental system. (b) Photograph and detailed structures of the OCA chip.

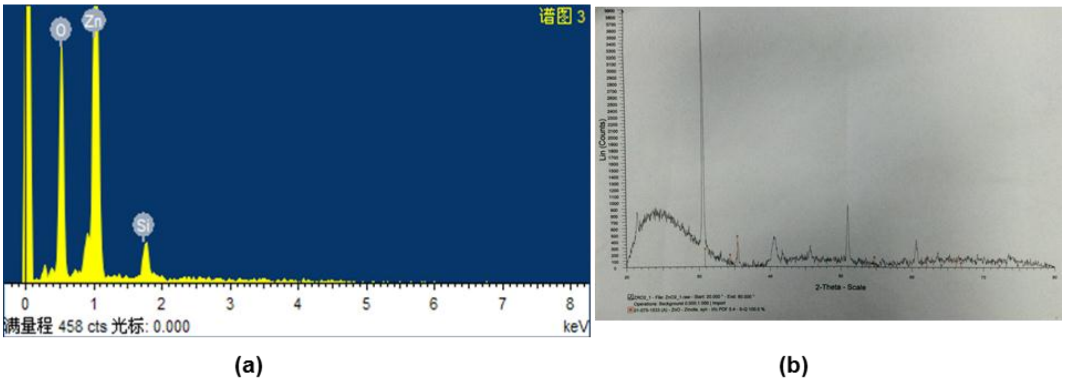


**Figure s2.** (a) XDS spectrum of deposited ZnO films, (b) XRD spectrum of deposited ZnO films.

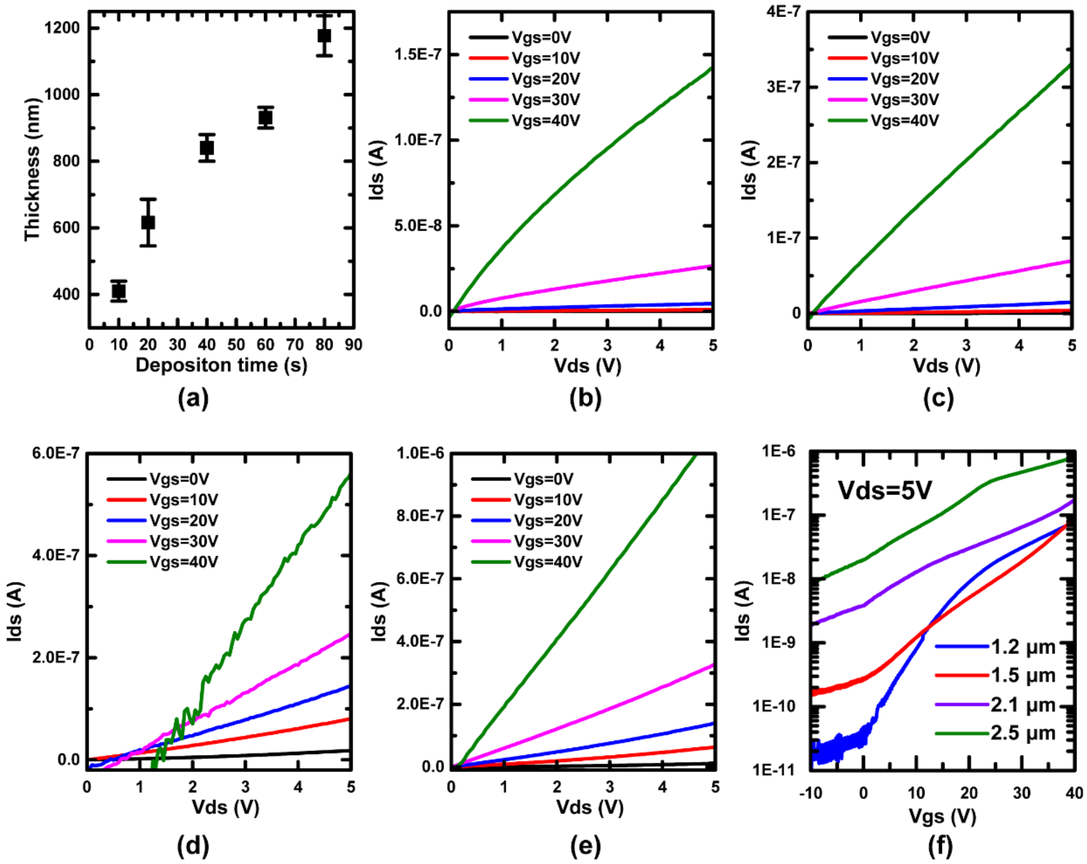


**Figure s3**. (a) Relationship between the thickness of ZnO films and the deposition time. (b)-(e) Output characteristic curves of TFTs with different thicknesses ZnO film: (b) 1.2 μm, (c) 1.5 μm, (d) 2.1 μm, and (e) 2.5 μm. (f) Transfer characteristics of TFTs with different thicknesses ZnO film.

***Note 2: Preparation of SWNTs solution***

The SWNTs solution used for assembling devices is prepared as following steps[1]: (1) dilute the bought SWNTs solution (1 % wt, from Chengdu institute of chemistry, China) with deionized water at a ratio of 1:4. (2) Sonicate the solution for 1 hour at a frequency of 59 kHz in a ultrasonication oscillator to obtain well-dispersed SWNTs

solution. (3) Centrifuge the solution for 1 hour at a speed of 3000g and a temperature of 4 ℃, and keep the bottom 70% solution for later process. (4) Centrifuge the left 70% solution for another 1 hour at a speed of 30000g and a temperature of 4 ℃, and keep the upper 50% solution for device manufacturing.

***Supplemented Video***

**Video S1**: Time-lapse process for patterning gold films

**Video S2**: Time-lapse process for patterning coper films

**Video S3**: Time-lapse process for patterning silver films

1 Xu, K., Dong, Z., Tian, X., Liu, J. & Wu, C. Assembly and Fabrication of Single-Wall Carbon Nanotube Field Effect Transistor Using Dielectrophoresis Method. *Integr Ferroelectr* **127**, 21-27 (2011).
